# Estrogens and Neuropeptides in Postmenopausal Women: Un Update

**Published:** 2012-04-30

**Authors:** M. Guida, F. Zullo, B. Buonomo, M.L. Marra, V. Palatucci, R. Pascale, F. Visconti, G. Guerra, ML Spinelli, A. Di Spiezio Sardo

**Affiliations:** 1 Department of Obstetrics& Gynecology, University of Salerno, Italy; 2 Health Sciences Department, University of Molise, Italy; 3 Department of Gynecology and Obstetrics, and Pathophysiology of Human Reproduction, University of Naples "Federico II", Italy

**Keywords:** estrogens, kisspeptin, neuropetide Y, neurokinin B, menopause

## Abstract

Menopause is characterized by depletion of ovarian follicles, a reduction of ovarian hormones to castrate levels and elevated levels of serum gonadotropins from the anterior pituitary gland. Although this process has significant repercussions throughout the body and affects a large proportion of our society, the neuroendocrine control mechanisms that accompany menopause are poorly understood. This review aims to examine rigorously the most accredited literature to provide an update about our current understanding of the role of the hypothalamic-pituitary axis in the onset of and transition into female reproductive senescence, focusing on the role of some specific neuropeptides in regulating the HPG axis and on their effects on several menopausal symptoms, especially referring to the cardiovascular risk, to open up new horizons for new therapeutic strategies.

## 1- Introduction

Menopause is a condition caused by the depletion of ovarian function followed by cessation of menstrual flow lasting at least 12 months. Modern medicine has significantly prolonged the lifespan of humans and most women spend one-third to half of their lifetime in post- menopause [1]. During the transition from reproductive to non-reproductive years, women often experience some psychological, somatic, vasomotor, and urogenital symptoms which may affect their daily activities [2]. In recent years, studies have shown that menopausal symptoms may affect health-related quality of life [3,4]. Ovarian aging and hormonal changes have been consistently linked to vasomotor symptoms, such as, hot flashes, night sweats, and some urogenital symptoms [5]. The causes of other symptoms reported by women in the menopausal transition are multifaceted ; moreover it is unclear to what degree these are related to aging generally or other life circumstances corresponding with menopause. Recent reports have also linked musculoskeletal pain, headaches, depressed mood, and perceived stress to menopausal stage and changes in reproductive hormones [6].

The reproductive neuroendocrine axis in postmenopausal women is intact and responds robustly (intensely) to the removal of ovarian hormones.

In primates, menopause is the signal of the complete collapse of ovarian function [7].

Decreased ovarian function is accompanied by the loss of circulating estradiol and progesterone, reduced steroid feedback and signaling to hypothalamic areas that modulate gonadotropin releasing hormone (GnRH) production and release and increased pituitary gonadotropin levels (FSH and LH) [8].

The gonadotropin releasing hormone (GnRH) system is the primary hypothalamic regulator of reproduction and extensive studies have demonstrated the role of the GnRH system in age-related reproductive failure.

This review wants to underline the role of kisspeptin, neurokinin B (NKB), calcitonin gene related peptide (CGRP) and several other neuropetides in female reproductive aging and some menopausal symptoms, mediating negative steroid feedback within the HPG axis and directly acting on different organs and tissues throughout the body.

## 2- Materials and methods

This review includes medical papers published in the English language since 1990 about the relationship between estrogens, some neuropeptides and HPG axis in pre, peri and postmenopausal women and their supposed role in menopausal symptoms, making particular reference to their effects on nasal mucosa and cardiovascular risk. All pertinent articles have been identified through a MEDLINE, PMC and EMBASE search and then reports have been selected through systematic review of all references.

## 3- HPA axis in human female reproductive aging: role of GnRH and neuropeptides

Despite the continued aging of the central nervous system, there is compelling evidence that many aspects of reproductive neuroendocrine function remain intact after menopause.

After the menopause, there are age-related changes in the reproductive neuroendocrine axis: studies using indirect pharmacological methods provide evidence that GnRH secretion is increased in postmenopausal women compared to premenopausal women [9]. Importantly, the ability of estrogen feedback to decrease GnRH secretion and gonadotropin secretion is not diminished by age [10,11].

The hypothalamus has been shown to be the major site of steroid negative feedback in the human [11,12].

These studies demonstrate intact hypothalamic function in the postmenopausal period and indicate that removal of steroid negative feedback in postmenopausal women is linked to increased GnRH secretion from the hypothalamus. Moreover, GnRH mRNA is increased in the hypothalamus of postmenopausal women, as would be expected with removal of steroid negative feedback [13]. The elevation of GnRH gene expression occurs within a subpopulation of neurons scattered in the ventral preoptic region, retrochiasmatic area and the infundibular nucleus but not within the dorsal preoptic area or septal region [13]. Combined with these studies, it has been also observed a pronounced enlargement of neurons in the hypothalamic infundibular (arcuate) nucleus of postmenopausal women [14, 15]. This cellular hypertrophy is characterized by increased Nissl substance (indicative of increased protein synthesis) and enlarged nuclei and nucleoli, suggesting increased neuronal activity [15, 16]. The neuronal hypertrophy does not appear to be a compensatory response to cell degeneration because there is neither cell loss nor signs of a pathological process in the infundibular nucleus of older women [15, 17].

Hybridization histochemistry studies have shown that the hypertrophied neurons express estrogen receptor α (ERα) and neurokinin B (NKB), kisspeptin (KiSS-1) and other neuropeptides’s mRNA and that menopause is associated with a striking increase in these neuropeptides’s gene expression [16, 17]. Remarkably, ovariectomy of young cynomolgus monkeys produces identical changes [18], providing strong evidence that the hypertrophy and increased NKB gene expression in postmenopausal women is secondary to the ovarian failure of menopause.

It has been proposed that these hypertrophied neurons participate in the hypothalamic circuitry regulating estrogen negative feedback, because the hypertrophy occurs in a subpopulation of Erα mRNA-expressing neurons in concert with estrogen withdrawal and gonadotropin hypersecretion [19].

### 3.1- History

Studies pioneered by the endocrinologist Selmar Aschheim in 1964 showed that ovaries transplanted from young female rats with normal estrous cycles into old, non-cycling mice failed to restore estrous cyclicity, suggesting that the ovary is not the sole determinant of female reproductive senescence [20]. In 1972, Peng and Huang reported that ovaries harvested from old mice and transplanted into young ovariectomized mice supported normal estrous cycles and formed corpora lutea, whereas hypothalamic transplants from old mice into young, ovary intact females did not [21]. These were the first studies to demonstrate clearly that exhaustion of ovarian gametes is not the exclusive determinant of female reproductive aging and they provided the first clue that the HPA is involved in female reproductive senescence.

More than four decades ago, Sheehan and Kovacs described pronounced differences in hypothalamic neuronal morphology between pre- and postmenopausal women [22]. The neurons were larger in postmenopausal women, in a subregion of the infundibular (arcuate) nucleus which they named the subventricular nucleus [22,23]. The enlarged neurons exhibited other signs of hypertrophy, including increased nuclear size, larger nucleoli and prominent Nissl substance. There was no evidence of increased storage material, chromatolysis, swelling or any other pathological changes that explained the change in neuron size. The hypertrophied neurons were identified in women over the age of 50 and in women with a history of post-partum hypopituitarism, but were inconspicuous in men of any age [22]. Sheehan proposed that the hypertrophy of neurons in postmenopausal women was related to loss of ovarian estrogen secretion, because the neuronal hypertrophy was strongly correlated with uterine atrophy in patients with post-partum hypopituitarism [24]. Subsequent analysis using computer microscopy showed a 30 to 40% increase in the size of neurons in the infundibular nucleus of postmenopausal women [15,17]. Stereological studies revealed no neuronal cell loss in the infundibular nucleus of older women [15]. Thus, the neuronal hypertrophy is not a compensatory response to adjacent neuronal cell death.

The development of *in situ* hybridization allowed characterization of mRNA expression in the hypertrophied neurons of postmenopausal women.

The hypertrophied neurons express ERα mRNA but do not express GnRH [17]. The increase in GnRH gene expression in postmenopausal women occurs in a separate subpopulation of neurons scattered diffusely in the ventral hypothalamus [13].

Hybridization of hypothalamic sections with a variety of cDNA probes revealed that the majority of hypertrophied neurons express, kisspeptin, neurokinin B (NKB), substance P (SP) and other tachykinins gene transcripts [16]. Thus, the hypertrophied neurons express two mRNAs that are essential for reproduction, NKB and kisspeptin [25, 26, 27].

Dynorphin mRNA is also expressed in the hypertrophied neurons, but unlike the elevation of NKB and kisspeptin gene expression in postmenopausal women, the expression of the mRNA encoding dynorphin is reduced [28].

Similar to the human, menopause in monkeys is characterized by ovarian failure and gonadotropin hypersecretion [29, 30, 31]. Quantitative PCR has shown increased kisspeptin and kisspeptin receptor mRNA in the medial basal hypothalamus of postmenopausal monkeys and these changes are duplicated by ovariectomy of young rhesus monkeys [32]. NKB gene expression is also increased in the arcuate nucleus of perimenopausal rhesus monkeys with low estrogen levels, and in monkeys after long-term ovariectomy [33]. Thus, as in women, NKB and kisspeptin gene expression is increased in the infundibular/arcuate nucleus of aging female rhesus monkeys, and these changes are likely due to the loss of ovarian steroids.

### 3.2- The KNDy Cells

In 2007 [34], a key observation was made when both NKB and kisspeptin, along with a third peptide, dynorphin (DYN), were shown to be colocalized in a single subpopulation in the hypothalamic arcuate nucleus (ARC) of the sheep.

DYN is an endogenous opioid peptide (EOP) that appears to mediate the inhibitory feedback control of progesterone on GnRH secretion [35, 36]. In postmenopausal women, dynorphin mRNA is decreased in the infundibular (arcuate) nucleus [28], consistent with the decrease in dynorphin gene expression reported in ovariectomized ewes [36]. In the mouse, however, estradiol has been shown to suppress dynorphin gene expression in the arcuate nucleus [37, 38]. Thus, a single subpopulation of neurons in the ARC contains three distinct neuropeptides, each of which has been strongly implicated in the feedback regulation of GnRH neurons; coexistence of multiple peptides in a single neuron is a common finding in the central and peripheral nervous system [39]. Typically, two or more peptides are stored together in large dense core vesicles and may be differentially released depending on their relative synthesis [39]. Changing the balance among the relative amounts of peptides could modify the reproductive axis. For istance, an increase in the gene transcription of excitatory peptides (NKB and kisspeptin) combined with a decrease in the transcription of an inhibitory peptide (dynorphin) could underlie the increased gonadotropin secretion in postmenopausal women [13,16,28, 40,41].

To simplify the reference, the name of this cell group has been abbreviated as the KNDy (coexpressing kisspeptin, NKB, and DYN) subpopulation; each of the component neuropeptides (kisspeptin, NKB, and DYN) when examined in dual-label studies showed a very high degree of colocalization with gonadal hormone steroid receptors, specifically the α-isoform of the estrogen receptor (ERα) [42, 44, 45, 46], the progesterone receptors (PR) [47] and the androgen receptor [48, 49].

Although each of these three peptides is found in separate sets of neurons in other brain regions, recent evidence suggests that the colocalization of KNDy peptides seen in the ARC is unique among brain regions and is conserved across multiple mammalian species that include the rat [42], mouse [38], sheep [34] and goat [43]. Complementary studies suggest that kisspeptin, NKB, and DYN are also colocalized in a single subpopulation in the human infundibular (ARC) nucleus [40]. These anatomical observations strongly suggest that KNDy cells constitute a conserved, central node in the control of GnRH secretion, playing a key role in normal physiological control of reproduction as well as in abnormalities leading to reproductive endocrine disorders.

Although we have defined KNDy cells on the basis of the presence of three peptides, this subpopulation may well contain other neurotransmitters as well as other receptors.

In the case of NKB/kisspeptin/dynorphin neurons, that neurotransmitter may be glutamate, based on the colocalization of vGLUT-2- immunoreactivity in the rat [50] and sheep [51].

### 3.3- The KNDy Cellsnetwork in the arcuate nucleus and projections to GnRH terminals in the median eminence

It’s necessary to show that KNDy cells directly contact GnRH neurons to understanding their functional role. Because colocalization of the three KNDy peptides in the ARC appears to be unique among brain areas examined to date, colocalization of multiple KNDy peptides in the same axon terminal can be used to determine the efferent targets of this subpopulation. Using this approach, it has been seen that NKB fibers project from the arcuate nucleus to both the internal and external zones of the median eminence, including the lateral palisade zone, a site with dense GnRH terminals: KNDy cells would provide direct inputs to GnRH neurons in the POA as well as in the mediobasal hypothalamus (MBH) at the level of their cell bodies and dendrites [52, 53].

One of the most interesting features of KNDy cells is that most appear to receive input from other KNDy cells and thus form a reciprocally interconnected network within the ARC [42, 47, 53]: arcuate NKB/kisspeptin/dynorphin neurons project extensively within the arcuate nucleus and extend across the median eminence to the contralateral arcuate nucleus [53]. Within the arcuate nucleus there is a dense network of NKB/dynorphin axons and close apposition of these axons on NKB/dynorphin cell bodies and dendrites, indicative of communication between these neurons [42]. It has been hypothesized that the KNDy network may play a role in the generation of episodic GnRH [38, 54] as well as in the coordination/amplification of responses to internal and external signals that KNDy cells are attuned to (*e.g*. gonadal steroids). In either case, one would predict that appropriate postsynaptic receptors to KNDy peptides should also be expressed within KNDy cells. Actually, this appears to be true for at least two of the three KNDy peptide receptors: the high-affinity receptor for NKB (NK3R) is colocalized in KNDy cells in rats [52], mice [38] and sheep [55] and the κ-EOP receptor (KOR), the opioid receptor subtype with highest specificity for DYN, is expressed within KNDy neurons in the mouse [38]. Although the kisspeptin receptor (*Kiss1r*) is expressed predominantly in GnRH cells, it is also found in other hypothalamic regions but, at least in mice, is not expressed in the ARC nucleus, suggesting a lack of colocalization within KNDy cells [56].

### 3.4 - Role in Generation of Episodic GnRH Secretion

The observation that KNDy neurons form an interconnected network presumably capable of producing a synchronous burst of firing has led to speculation that they may represent an important component of the multiunit activity known as the GnRH pulse generator [38, 43]. It has been proposed that synchronous activity of KNDy neurons is controlled by stimulatory actions of NKB and inhibitory actions of DYN on these neurons and that their output to GnRH neurons is primarily via kisspeptin. Kisspeptin neurons express ERa, PR and AR and therefore have the potential to relay feedback effects on the GnRH neuron [40]. Actually evidence suggests that reduced activity of kisspeptin neurons in the Arc of rodents, primates and sheep is responsible for translating estrogen negative feedback to GnRH neurons. OVX female and castrated male mice [46, 49], sheep [57, 58] and rhesus monkeys [41,59] have an increased level of KiSS-1 mRNA in the neurons compared with controls. Moreover, if estrogen replacement is given to OVX female or testosterone is given to castrated male mice, sheep and rats, then KiSS-1 mRNA levels are reduced to control levels [41, 49, 57, 58,59, 60]. This suggests that steroids are negatively regulating KiSS-1 mRNA in the Arc, hence reducing stimulation of GnRH neurons. Further evidence for negative feedback is derived from females at the time of menopause, when estrogen is low due to reduced follicle numbers in humans and rhesus monkeys. At this time, a rise in KiSS-1 mRNA and in turn LH is seen accompanied by cellular hypertrophy, similar to the rise in OVX female rhesus monkey [32, 40,41], which is thought to be due to the lack of negative feedback from the follicles.

Each GnRH pulse is triggered by an initial increase in NKB from a few KNDy neurons, which stimulates further NKB release; the resulting positive feedback loop produces release of kisspeptin onto GnRH neurons and hence the extremely rapid increase in GnRH secretion that occurs at the onset of a pulse [61]. NKB stimulation of KNDy neurons is predicted to also stimulate release of DYN, and the inhibitory actions of this EOP on KNDy neurons first begins to hold kisspeptin release in check and, after a few minutes, completely suppresses the activity of KNDy neurons, terminating the GnRH secretory episode and preventing any GnRH secretion between pulses. The action of DYN on KNDy neurons will also suppress DYN release, which eventually allows increased firing and the NKB release that triggers the next GnRH pulse. Thus, we speculate that kisspeptin in KNDy cells is the output that drives GnRH pulses, that NKB is the trigger that initiates synchronous firing of KNDy neurons and the onset of each pulse, and that DYN is the peptide that shuts off the firing of KNDy neurons and terminates each pulse [62]. Although this model must be rigorously tested, it does provide a simple explanation for the differential roles of kisspeptin and DYN in the negative feedback actions of E_2_ and progesterone, respectively:because kisspeptin release from KNDy neurons is driving GnRH release during a pulse, E_2_ inhibition of kisspeptin expression would be expected to inhibit GnRH pulse amplitude. In contrast, DYN terminates and prevents GnRH secretion between pulses. Thus, a stimulation of DYN release by progesterone might be expected to prolong the interval between pulses and thus reduce GnRH pulse frequency.

There is compelling evidence that changes in KNDy cells occur as a part of the process of normal aging in association with menopause. Specifically, it has long been known that in the brains of postmenopausal women, there is selective hypertrophy of neurons of the infundibular (arcuate) nucleus of the human hypothalamus [14]. Rance and colleagues have shown in single-label studies that the large majority of these hypertrophied neurons each contain *KISS1*[41], NKB [16], and DYN [28] as well as ERα [17]mRNA; thus, they likely represent KNDy cells of the human hypothalamus. In postmenopausal women, there is increased gene expression of NKB [16] and *KISS1*[41] in these cells, along with decreased gene expression of DYN [28], consistent with an alteration in the balance between stimulatory (kisspeptin and NKB) and inhibitory (DYN) KNDy peptides that would lead to the GnRH and LH hypersecretion characteristic of postmenopausal women [40]. These changes in postmenopausal women are a response to the ovarian failure and depletion of ovarian steroids that occurs during menopause, because similar changes in KNDy peptide gene expression are seen in young OVX monkeys [40].

## 4 – Neuropeptides, estrogens and cardiovascular risk in postmenopausal women

Cardiovascular disease (CVD), such as coronary artery disease (CAD) and hypertension (HTN), is more common in men than in premenopausal women (Pre-MW) of the same age, suggesting cardiovascular benefits of estrogen [63, 64]. Epidemiological studies have shown that death due to CAD is delayed by ∼5 years in Pre-MW as compared to men [65]. Moreover, Pre- MW are 4–5 times less likely than men to have ischemic heart disease [66]. With aging the incidence of CVD becomes higher in women than in men. The increased risk of CVD in postmenopausal women (Post-MW) has been linked to the decrease in plasma estrogen levels, thus prompting further investigation of the effects of estrogen on the cardiovascular system. In this section of our review, we want to underline a possible role of some neuropepides in several menopausal symptoms and, above all, their influence on CVD risk in Post-MW, starting fromstudies about the relationship between nasal symptomatology and function and local concentration of estradiol and its receptor and neuropeptides (VIP, NPY and SP) in postmenopausal women complaining of “*paradoxical nasal stuffiness*” (i.e. sensation of nasal blockage without swelling and/or anatomical alterations) (Nappi et al., 2003). After this study, the effects of menopausal status on the content of vasoactive neuropeptides in the arterial wall were investigated (Di Carlo et al., 2005).

Then, we analize recent studies about the effect of two neuropeptides (CGRP and orexin-A) on CVD in post-MW [67, 68] and, at last, the outcomes of hormonal replacement therapy (HRT) [69].

### 4.1 - Nasal mucosa, estrogens and neuropeptides in Post-MW

Postmenopausal women often complain of reduced nasal function, both in terms of olfactive deficit and nasal blockage, even if no swelling or morphological alterations can be demonstrated [70]. Elderly subjects show a generalized decrease in body water content and the degeneration of mucus-secreting cells determining a reduced effectiveness of the mucociliary system with frequent symptoms of nasal stuffiness [70]. Moreover, recurrent epistaxis is also common after the menopause [71].

The activity of nasal mucosa in women is supposed to be related to variations in sex hormones[72,73]. Indeed, different histochemical and ultramicroscopical studies of this tissue have been performed, observing variations during the menstrual cycle [74], pregnancy [75], oral contraceptive use [76] and after menopause [77]. Estrogens may modulate nasal mucosa function by affecting the basal vasculature and the glandular secretion either directly or indirectly by modulating the local concentration of neuropeptides and their receptors [78]: both vascular tone and glandular secretion in the respiratory nasal mucosa are controlled by a complex network of regulatory neuropeptides. Among these substances, a prominent role is exerted by parasympathetic peptides, such as vasoactive intestinal peptide (VIP), sympathetic peptides, such as neuropeptide Y (NPY), and a group of peptides released by sensory nerve endings, known as sensory neuropeptides, such as substance P (SP) [79].

Nappi et al. [80] demonstrated that postmenopausal hypoestrogenism increases vasoconstrictor neuropeptides (NPY) and decreases vasodilator neuropeptides (VIP and SP) content in nasal mucosa and probably this is the trigger for reduced nasal mucosa trophism and effectiveness of the mucociliary system with consequent paradoxical nasal stuffiness. After HRT SP, VIP, E2 and ER was significantly increased; therefore both SP and VIP seems to be under a positive estrogen control at the nasal level. Moreover, a significant decrease of NPY was observed after HRT. This could support the hypothesis that estrogens have an inhibiting effect on NPY at the nasal level [81]. In conclusion, HRT might exert a positive effect on nasal function in postmenopausal women complaining of paradoxical nasal stuffiness, that means estrogens have positive effects on nasal mucosa, directly and indirectly, modulating the local concentration of some vasoactive neuropeptides.

This study has been the starting point to investigate the effects of menopausal status on the content of vasoactive neuropeptides in the arterial wall.

### 4.2 - Neuropeptides content in arterial-wall autonomic terminations in Post-MW

Autonomic pathways play a critical role in regulating vascular resistance by allowing selective redistribution of blood flow to those parts which have a greater demand [82]. Cathecolamine-synthesizing vasoconstrictor neurons also express neuropeptide Y (NPY), while autonomic vasodilator neurons, besides synthesizing acetilcholine, utilize vasoactive intestinal peptide (VIP) and substance P (SP) [82].

From January 2005–June 2005, Di Carlo et al. enrolled 20 premenopausal women (mean age, 49.7 years) and 20 postmenopausal women (mean age, 50.4 years), ≥12 months and ≤ 24 months after menopause, who were undergoing abdominal hysterectomy with bilateral oophorectomy for benign conditions to evaluate the presence of NPY, VIP, SP, E2, estrogen receptor α (ERα), and S100 (a generic neuronal marker used to assess the content of neuronal fibers) in the uterine arteries of pre- and post- menopausal women of similar age [83]. In Post-MW, a reduction in E2 and ERαin the uterine artery wall was associated with a decrease in SP and VIP, which are vasodilator neuropeptides, and with an increase in NPY, which is a well-known vasoconstrictor peptide [83]. No difference in S100 content was observed between the two groups so, the differences in neuropeptide content were likely the consequence of a functional shift, rather than being caused by an alteration in the number of neuronal fibers [83]. This study suggests that postmenopausal hypoestrogenism may cause an increase in arterial vascular tone through a reduction of vasodilator peptides and an increase in vasoconstrictor peptides in the arterial-wall termination of the autonomous system [83]. These changes in neuropeptide content in the arterial walls might represent a new mechanism underlying the negative effects of menopause on the cardiovascular system.

### 4.3 – Role of Calcitonin gene-related peptide and orexin-A in CVD risk in Post-MW

#### 4.3a – CGRP and CVD risk in Post-MW

Calcitonin gene-related peptide (CGRP), identified in 1982, belong to the calcitonin family of neuropeptides which also includes adrenomedullin (AM), amylin, intermedin, calcitonin and calcitonin receptor-stimulating peptide [84]. CGRP is a 37-aminoacid neuropeptide with widespread expression such as in the heart, blood vessels, pituitary, thyroid, lung and gastrointestinal tract and has a wide array of biological effects including neuromodulation, vasodilatation, cardiac contractility, bone growth, and mammalian development [84]. Potent subtype selective nonpeptide agonists and antagonists for CGRP are being developed for therapeutic use in hypertension, cardiac failure, migraine headaches, Reynaud’s syndrome, preeclampsia, and diabetes [84]. The role of CGRP to influence cardiovascular risk factor in postmenopausal women has been recently recognized. In one of the studies to determine the effects of menopausal status on circulating CGRP levels and the correlation between circulating CGRP levels and biomarkers for CVD, mean circulating CGRP levels were compared between Pre-MW and Post-MW: this showed CGRP levels higher in the postmenopausal women compared with premenopausal women [85]. Besides, serum CGRP levels positively correlated with serum insulin levels. These data show that circulating CGRP levels are influenced by menopausal status and suggest additional mechanisms through which the increased risk of hyperinsulinemia and CVD may arise in postmenopausal women [85].

In another study [86], it was observed the occurrence of CHD was correlated with high homocysteine (Hcy) level and low CGRP level. The mean serum CGRP level was significantly lower in postmenopausal women with CHD than in women without CHD. The mean serum Hcy level was significantly higher in CHD than in without CHD postmenopausal women. Hcy is an independent risk factor of CHD. *CGRP,* estradiol (E(2)) and progesterone (P) are independent protective factors of CHD. There was no relationship between Hcy, CGRP and E(2), and P [87].

Current evidences of role of molecule CGRP in pathophysiology of menopausal symptoms really stimulate us further for the assessment of role CGRP receptor antagonists such as olcegepant and telcagepant in ameliorating various postmenopausal symptoms.

#### 4.3b – Orexin-A and CVD risk in Post-MW

Orexins A and B (also known as hypocretins 1 and 2) are recently discovered hypothalamic neuropeptides, involved in the regulation of feeding behaviour, sleep–wakefulness, and neuroendocrine homeostasis [88, 89]. In addition to this central role of orexins as excitatory neurotransmitters, a putative peripheral effect has been suggested after the detection of substantial levels of orexins in plasma [90] as well as the demonstration of orexin receptors in several peripheral tissues, including the gastrointestinal tract (GIT), endocrine pancreas, adrenal glands, and adipose tissue, among others [91, 92]. Recent physiological and neuroanatomical studies suggest that orexin A may play an important role in the control of the hypothalamo–pituitary–gonadal axis [93] and gonadotropin-releasing hormone (GnRH) neurons in the hypothalamus have been found to be receptive to orexin modulation [94].

El-Seedek et al. have reported postmenopausal women not on ERT had significantly higher plasma orexin-A levels, paralleling the significantly lower estrogen levels. Arterial blood pressure was also significantly higher in this group [68]. It might be speculated that orexin A partially mediates this pressor response by: increasing basal sympathetic activity, causing catecholamine release [95], modulating the vasopressin system [96], and stimulating renal and adrenal orexin receptors [97]. These speculations are further justified by the study of Shiraska et al. [98], where experimental use of orexin A has been shown to increase heart rate, renal sympathetic activity, catecholamine release, and mean arterial blood pressure.

Postmenopausal women not receiving ERT had significantly higher plasma cholesterol and TG levels than reproductive-age women, but, more importantly, the levels were also higher than in those receiving ERT [68]. Orexins have been shown to adversely affect the plasma lipoprotein profile [99] and insulin glucose homeostasis, and to stimulate insulin release from pancreatic cells in vivo and in vitro [100].

It is also thought that orexins act in harmony with ghrelin, leptin and NPY, among others, to control energy balance and metabolism, and derangement in any of these can be associated with obesity [101].

The conclusion is that plasma orexin-A levels are elevated after menopause. The association between this elevation and hypoestrogenism was suggested when normal values were found in matched postmenopausal women on ERT. A putative causal relationship is suggested between increased orexin levels and some of the manifestations of the menopausal syndrome, notably, an increased cardiovascular risk profile so, a possible inhibitory effect of estrogen on orexin might partially account for its cardioprotective effect [68].

### 4.4 – HRT and CVD risk in Post-MW

Nowadays, whether menopausal hormone therapy (MHT) is beneficial in postmenopausal CVD remains controversial [102]. Despite reports of vascular benefits of MHT from observational and experimental studies, randomized clinical trials (RCTs), such as the Heart and Estrogen/progestin Replacement Study (HERS), the Women’s Health Initiative (WHI)and Raloxifene Use for The Heart (RUTH)have suggested that, contrary to expectations, MHT may increase the risk of CVD: in the HERS, almost 3,000 women with proven CHD were randomly assigned to MHT containing CEE and MPA or placebo. After 4 years, the frequency of the primary outcome, i.e. fatal and non-fatal CHD combined, did not differ between the two groups. There was also a 50% increase in coronary events in the first year in the MHT group [103]. The WHI was designed to determine fatal and nonfatal heart disease, cancer, and osteoporotic fractures as the primary outcome. In the estrogen-progestinarm, MHT increased risk of heart attacks and strokes. CEE alone increased risk of stroke and deep vein thrombosis [104]. The RUTH RCT in Post-MW with CVD or at increased risk of CVD demonstrated that raloxifene treatment did not change the incidence of coronary events, but increased the risk of fatal stroke and venous thromboembolism [105,106].

Bifulco et al. compared the effects of HRT and tibolone on nasal symptomatology/function and on local concentrations of estradiol and its receptor and neuropeptides (VIP, NPY and SP) in postmenopausal women complaining of paradoxical nasal stuffiness. All treated patients reported an improvement in climacteric symptomatology; theevaluation of the immunopositivity for E2, estradiol receptor (ER), Substance P (SP), Vasoactive Intestinal Peptide (VIP) and neuropeptide Y (NPY) revealed HRT induced a significant increase in E2, ERα, VIP and SP and a decrease in NPY immunopositivity. Tibolone determined a significant increase in ERα, VIP and SP and a decrease in NPY immunopositivity without any action on E2 immunopositivity so, both treatments may modulate nasal mucosa function through an action on cholinergic, adrenergic and sensory peptides and improve nasal function and symptomatology in postmenopausal women with paradoxical nasal stuffiness.

On the basis that the alterations seen in nasal mucosa of Post-MW with paradoxical nasal stuffiness may be a clinical model of the general effect of menopausal status on cardiovascular system, this study could suggest HRT could positively act on CVD risk, modulating the levels of vasoconstrictor (NPY) and vasodilator neuropeptides (VIP and SP).

## 5 – Discussionsand conclusions

This review demonstrates that a complex network between GnRH, FSH, LH, estrogens and several neuropeptides plays an important role in the transition into female reproductive aging and in the pathogenesis of some of the main menopausal symptoms (above all, the cardiovascular risk in Post-MW).

Understanding how the synthesis and release of individual KNDy peptide is orchestrated within KNDy neurons and their projections, and ultimately translated into control of the reproductive neuroendocrine axis, will likely have key relevance for a wide range of issues affecting reproductive health and disease.

Estrogen and other estrogenic compounds have significant vasodilator and other beneficial vascular effects. Some of the effects of estrogen are mediated via vascular ER. As things stand now, medical evidence-based guidelines and the FDA recommendations are that MHT may be used for the relief of moderate to severe postmenopausal symptoms, but not for the routine prevention of chronic disease conditions and primary or secondary Coronary Heart Disease (CHD), with the exception that MHT may be used conditionally for the prevention of osteoporosis when other interventions have been considered and are deemed inappropriate.

Nowadays, very little is known about the effects of estrogens on autonomic vasoconstrictor and vasodilator neuropeptides at the arterial level The report of a tight correlation between postmenopausal hypoestrogenism and the content in arterial-wall autonomic terminations of different vasoactive neuropeptides (such as NPY, SP and VIP) open up new horizons for the comprehension of the biology of menopausal status and for the finding of new therapeutic strategies to treat several menopausal symptoms and reduce the menopausal CVD risk.
